# Effects of different processing methods on the functional, nutritional, and physicochemical profiles of cowpea leaf powder

**DOI:** 10.1111/1750-3841.17569

**Published:** 2024-12-15

**Authors:** Makafui Borbi, Lorraine Weatherspoon, Jason Wiesinger, Jose Jackson, Raymond Glahn, Leslie Bourquin, Kirk Dolan

**Affiliations:** ^1^ Department of Food Science and Human Nutrition Michigan State University East Lansing Michigan USA; ^2^ USDA‐ARS, Robert W. Holley Center for Agriculture and Health Ithaca New York USA

**Keywords:** cowpea leaves, dehydration, heavy metals, minerals, physicochemical properties

## Abstract

Indigenous fruits and vegetables can improve food security and biodiversity. However, their use is hindered by perishability, seasonal availability, cooking losses, lack of nutritional composition data, and connections to low socioeconomic status. This study aimed to process cowpea leaves into powder and determine the effect of five home‐cooking methods on their protein, functional, physicochemical, and heavy metal profiles. Cowpea leaves were boiled, blanched, steamed, sous‐vide cooked, and stir‐fried, at 5, 10, and 15 min before dehydration at 60°C. Cowpea leaves contain protein up to 20 g/100 g. The leaves are rich in calcium, potassium, and zinc, providing up to 70% of the adult recommended dietary allowance for calcium and potassium per 100 g of powder. Cowpea leaf powder exhibited good water/oil absorption and rehydration capacities. Sous‐vide and steamed cowpea leaves provided an overall superior nutritional profile (*p* ≤ 0.05). Heavy metals in the cowpea leaf powders were below the WHO permissible limits except for aluminum and high arsenic levels. This study demonstrated that cowpea leaf powders could be potentially incorporated into foods to improve functional properties and nutrient intake.

## 1 INTRODUCTION

African indigenous vegetables, often considered neglected treasures, are underutilized yet adaptable to extreme climate conditions and contain significant amounts of proteins, fiber, essential micronutrients, and phytochemicals, thus holding potential for enhancing food and nutrition security as well as biodiversity (Mungofa et al., 2022). Despite hundreds of plant species being consumed in Africa, there are gaps in knowledge regarding complete nutritional data, the effects of cooking/processing, appropriate preservation methods, and their potential for more extensive inclusion in the diet. Challenges, such as seasonal availability and short shelf‐life, hinder their extensive utilization. Misconceptions persist, such as the belief that they are only consumed by those experiencing poverty and concerns about contamination with heavy metals (Misci et al., 2022; Moyo et al., 2021; Mungofa et al., 2022).

The interest in indigenous foods has garnered renewed global attention in recent years, driven by factors such as climate change, rapid population growth, and increasing diet‐related diseases. Initiatives like the Vision for Adapted Crops and Soils program, a partnership between the African Union Commission, the US Department of State, and the Food and Agriculture Organization (FAO), aim to develop resilient food systems based on diverse, nutrient‐dense, climate‐adapted crops grown in healthy soils. Similarly, national and international programs, like the Periodic Table Food Initiative and the USDA Indigenous Food Sovereignty Initiative, have received substantial funding to explore the role of indigenous foods in food and nutrition security (Ahmed et al., 2022).

To promote local vegetable consumption for food and nutrition security, it is essential to identify and characterize nutrient‐dense indigenous vegetables and fruits, focusing on preservation, cooking methods, and integration into the diet (Owade et al., 2021). Further research is needed to establish a comprehensive database of underutilized indigenous foods and key considerations for use.

The leaves of cowpea (*Vigna unguiculata*), also known as the black‐eyed peas, are easy to cultivate and are a good source of protein and micronutrients (Okonya & Maass, 2014). Dehydrated leaves contain high concentrations of iron, vitamin A, calcium, and zinc. Cowpea leaves can provide up to 25% and 75% recommended dietary allowance of iron and vitamin A, respectively, for 4‐ to 8‐year‐old children (Owade et al., 2020). Despite their nutritional value, fresh cowpea leaves are still underutilized across Africa because of their short shelf‐life (up to 48 h) and seasonal availability (Owade et al., 2020). In Ghana, for example, cowpea leaves are mostly used in only the Northern regions. The leaves are typically consumed after boiling for extended periods, alone, or in soups and stews. Traditional cooking and preservation methods like boiling, sun‐drying, and fermentation have been explored but may compromise nutritional and sensory qualities (Owade et al., 2020).

Various cooking methods, including boiling, steaming, frying, and sous vide, can significantly impact the color, texture, and nutrient retention of vegetables. Mineral losses can occur due to leaching or heat application (Barciela‐Alonso & Bermejo‐Barrera, 2015). Factors such as cooking temperature, time, and exposure to water or oil influence these changes (Jiang et al., 2023). Blanching effectively inhibits browning, whereas sous vide, due to minimal water contact and lower temperatures, reduces nutrient loss (Cui et al., 2021). Stir‐frying has been shown to enhance greenness in kale (Akdaş & Bakkalbaşi, 2017). In Ghana, traditional methods like boiling predominate, but exploring techniques, like sous vide and stir‐frying, could guide consumers to select methods that retain physical qualities and nutrients in cowpea leaves. Additionally, pre‐cooking before dehydration offers a way to incorporate nutritious leaf powders into ready‐to‐eat foods (Owade et al., 2020).

This study aimed to determine the effects of blanching, steaming, sous vide, and stir‐frying on protein, selected functional properties, and mineral and heavy metal profiles of cowpea leaves and to compare with traditional methods such as boiling. To the best of authors’ knowledge, this is the first study to investigate the impacts of sous‐vide cooking on cowpea leaves.

## 2 MATERIALS AND METHODS

### 2.1 Materials

Fresh cowpea leaves were purchased from a local market in the Accra region and the Northern region of Ghana. The raw materials were purchased from at least 10 vendors in different markets to ensure representative sampling. The procured materials were transported to the Food and Research Institute in Accra for processing.

### 2.2 Processing of cowpea leaves

Cowpea leaves were sorted to eliminate immature, damaged pieces for uniformity. The raw materials were cleaned, washed, and rinsed in 1% saline water followed by potable water. Cowpea leaves were divided into 1 kg five batches—one for each processing method. Boiling, blanching, steaming, sous vide, and stir‐frying. Hot water blanching was done at 100°C in stainless steel pots, and then rapidly cooled. For sous vide, leaf samples were vacuum sealed at 25 mbar (0.4 psi) and cooked in a water bath at 80 ± 2°C for 5, 10, and 15 min. Stir‐frying was done with vegetable oil (Frytol brand, a blend of rapeseed, palm oil, and sunflower oil) according to the method by Wu et al. (2021), using 10 mL oil per 100 g of leaf samples. For boiling, the leaves were placed in a stainless‐steel cooking pot and boiled in water for 30 min. Three processing times were used for each method (5, 10, and 15 min).

Pretreated cowpea leaves were dehydrated in a cabinet air dryer at 60 ± 2°C for 5–6 h with 60% relative humidity, to a final moisture content of ∼15%. Dried samples were milled using a heavy‐duty electric spice grinder (VEVOR XZ‐10B). Cowpea leaf powder (CLP) was cooled, packed, and sealed in 3‐mil polyethylene bags and stored at −20°C until analyzed.

### 2.3 Functional properties

For functional properties, the powders were sieved with a 250 µm sieve before analysis to eliminate the effect of particle size and ensure a fair comparison of vegetable powders and processing methods.

#### 2.3.1 Bulk density

Bulk density (BD) was determined by the method of Waseem et al. (2021). Briefly, 50 g of each sample was poured into tarred 100 mL graduated cylinders. The cylinder was tapped gently twice to fill any remaining space for homogeneity, and the BD was estimated using the formula:

$$\mathrm{BD}=\frac{\mathrm{Sample}\;\mathrm{weight}\;\left(g\right)}{\mathrm{Volume}\;\mathrm{occupied}\;\left(\mathrm{mL}\right)}$$

#### 2.3.2 Water absorption capacity (WAC) and oil absorption capacities (OAC)

The water absorption capacity (WAC) of the samples was estimated using the method followed by Waseem et al. (2021). One gram sample was mixed in 10 mL of distilled water (WAC) or canola oil (specific gravity = 0.92) for oil absorption capacity (OAC). The mixture was left to stand for 30 min at room temperature 25 ± 2°C and centrifuged (Sorvall RC‐5B, Thermofisher) at 2000 rpm for 10 min. The supernatant was decanted, and the residues were drained on filter paper for 5 min. WAC and OAC were determined by weighing the residues to find the amount of water (g) absorbed per gram of powder.

#### 2.3.3 Water‐solubility index (WSI)

The water‐solubility index (WSI) was determined according to the method of Waseem et al. (2021). One gram of the sample was mixed with 10 mL of distilled water. The mixture was centrifuged at 4000 rpm for 30 min using refrigerated centrifuge (Sorvall 6B, DuPont Co.). The supernatant was dried in an air‐forced moisture oven at 105 ± 2°C overnight to a constant weight. Dried samples were cooled, weighed, and WSI calculated using the following formula:

$$\mathrm{WSI}=\frac{\mathrm{Weight}\;\mathrm{dried}\;\mathrm{supernatant}\;\left(g\right)\;}{\mathrm{Weight}\;\mathrm{original}\;\mathrm{sample}\;\left(g\right)}\times 100$$

#### 2.3.4 Rehydration ratio

The rehydration ratio was carried out using the method used by Waseem et al. (2021). Five grams of each sample were soaked in 50 mL of distilled water for 60 min at room temperature. Soaked samples were filtered using Whatman filter paper No. 41. The permeate was weighed, and the samples’ ability to rehydrate upon soaking was measured using the formula:

$$\mathrm{Rehydration}\;\mathrm{ratio}=\frac{\mathrm{Weight}\;\mathrm{of}\;\mathrm{drained}\;\mathrm{material}\;\left(g\right)}{\mathrm{Weight}\;\mathrm{of}\;\mathrm{dried}\;\mathrm{residues}\;\left(g\right)}$$

#### 2.3.5 Dispersibility

Dispersibility was measured according to the method of Elkhalifa et al. (2017). Briefly, 10 g of the sample was weighed into a 100‐mL glass cylinder. Distilled water was added to make the volume 100 mL, stirred vigorously, and allowed to settle for 3 h. The volume of settled particles was subtracted from 100, and the difference was reported as percent dispersibility.

#### 2.3.6 Particle size distribution

The mechanical sieve analysis method (Lyu et al., 2021) was used to conduct a particle size distribution analysis using pore sizes classified in this study as coarse (600 and 500 µm) and fine (250 and 180 µm). All samples were analyzed except stir‐fried samples that lacked flowability and were more cohesive due to oil content and could not be sieved. These four sieves were stacked and loaded on an Orbital Sieve Shaker (Cole‐Parmer SSH‐200‐SS‐15D, Cole‐Parmer) in descending order with the pan at the bottom. For each sample, 50 g was weighed into the 600‐µm sieve and shaken for 10 min. The sample size of each sieve and pan was collected and weighed. Cumulative passing was calculated and reported as a percent of the total sample that passed through each sieve.

### 2.4 Physicochemical properties

#### 2.4.1 Tristimulus color

The color of each sample was determined by the method reported by Pekmez and Yılmaz (2018). Objective color measurement was made based on three‐color coordinates, *L*, *a*, and *b* using Hunter Lab Mini‐Scan XE Plus colorimeter (Hunter Lab). Physical color L* represents brightness, a* (redness), and b* (yellowness). The instrument was calibrated with black and white reference tiles. Color measurements were done in triplicate, and average values were used for calculation.

#### 2.4.2 Moisture and water activity

The moisture content was measured using the AOAC method. Samples were weighed into aluminum pans and dried overnight in an oven at 105°C (Ren & Sun, 2022). The dried samples were weighed, and the moisture was reported as g/100 g of the original sample. Water activity (*a*_w_) was determined using a water activity meter at room temperature, 25°C.

#### 2.4.3 pH

The method reported by Wickramasinghe et al. (2020) was used pH analysis. A slurry was made with a 1:10 w/v ratio of sample to water, mixed thoroughly, and allowed to stand for 30 min. The pH was measured with an electronic pH meter (Oakton pH 150).

#### 2.4.4 Protein

Protein content was determined by the Dumas method using a nitrogen/protein analyzer (LECO, FP828, LECO Corporation) (Dee & Chang, 2024). Instead of the Jones factor of 6.25, a more conservative nitrogen‐to‐protein conversion factor of 4.5 accounted for the often‐overlooked non‐protein nitrogen in vegetables. This factor was based on recommendations for leafy greens like spinach and eggplant (Fujihara et al., 2001).

#### 2.4.5 Minerals and heavy metal analysis

Inductively coupled plasma atomic emission spectroscopy (ICP‐AES) (Thermo Scientific, iCAP 6500 series) was used for analysis according to the method described by Katuuramu et al. (2021) and Carboni et al. (2020). Samples (250 mg) were predigested in borosilicate glass tubes with 3 mL of concentrated high‐purity nitric and perchloric acid mixture (60:40 v/v) for 16 h at room temperature. They were then placed in a digestion block (Martin Machine) and gradually heated to 120°C over 4 h, incubated at 120°C for 2 h, with an additional 2 mL of nitric acid, and then heated to 145°C for another 2 h. The temperature was increased to 190°C for at least 10 min to evaporate the acid, then cooled to room temperature. Digested samples were resuspended in ultrapure water and analyzed using ICP‐AES with quality control standards (High Purity Standards). Yttrium (10M67‐1) was used as an internal standard.

### 2.5 Statistical analysis

All experimental work was done using three replicates, and the mean values were calculated and presented as mean ± standard deviation (SD). One‐way analysis of variance (ANOVA) technique was performed on data collected. The means were separated using Tukey's honestly significant difference at a confidence interval of 5%. Correlation analysis was performed to examine the relationship between the different functional properties and physicochemical variables measured. Pearson's correlation coefficient was determined at a 95% confidence interval to test the significance level.

## 3 RESULTS AND DISCUSSION

### 3.1 Functional properties

#### 3.1.1 Bulk density

The bulk density for CLP ranged from 0.34 g/mL for 5‐ and 10‐min sous‐vide CLPs to 0.47 g/mL for 10 and 15 min blanched CLs (data not shown). No significant differences were observed for bulk density across all cooking treatments. Our results differ from those reported by Mythili et al. (2021) for bulk density of cauliflower leaf powder, which was significantly higher in blanched samples than the control. The bulk densities for CLP in this study were lower than those reported for raw dried spinach leaves by Waseem et al. (2021). Factors, such as vegetable type, blanching, initial moisture content, and particle size, may contribute to these differences (Roongruangsri & Bronlund, 2016). Singh and Prasad (2013) noted that reducing particle size leads to lower bulk density due to increased interparticle pore size in Moringa powders. Similarly, Huang et al. (2020) showed reduced bulk density with superfine grinding of Moringa leaves after blanching, steaming, and dehydration. BD indicates the degree of porosity in a product, influencing the choice of packaging materials. Products with lower bulk density need lighter, less dense packaging, whereas those with higher bulk density require more robust, dense materials for proper containment (Roongruangsri & Bronlund, 2016).

#### 3.1.2 Water absorption capacity (WAC) and water‐solubility index (WSI)

The WAC of CLPs ranged from 1.77 to 4.26 g/g (Table 1). Samples fried in oil showed the lowest water absorption. This effect was most likely due to increased hydrophobicity from increased fat content. Mythili et al. (2021) found similar WAC values in underutilized cauliflower leaves and spinach powder, respectively.

Among all cooked samples, steamed cowpea leaves generally showed the least WAC, followed by sous vide. Boiled samples had the highest WAC, which were similar to blanched and raw cowpea leaves. Cooking time did not have a significant impact on WAC results. The general trend identified in our study is comparable to those obtained by Mythili et al. (2021), except we did not find a significant difference between blanched and raw cowpea leaves (Table 1). A positive correlation was observed between protein content CLPs and WAC (Pearson coefficient = 0.847**, significant at p<0.01) (Table S1) Proteins can absorb substantial amounts of water, but the opposing associations are likely due to the differences in protein types, structure, and amount of hydrophilic and hydrophobic amino acid content found in the cowpea leaves.

Methods that had minimal contact with water, that is, steaming and sous vide, improved water solubility, which increased with cooking time. However, Mythili et al. (2021) found that solubility decreased when blanching with hot water and steam. Differences in the two studies could be attributed to differences in particle size. In contrast to WAC, the relationship between WSI and protein was not significant. The WAC and WSI are both functions of particle size, protein, and carbohydrates and are important in bulking and consistency, especially in ready‐to‐eat food (Mohajan et al., 2018; Suriya et al., 2016). The addition of powders with high WAC as ingredients to food products may positively impact viscosity, texture, and consistency in baked goods (Bas‐Bellver et al., 2022).

#### 3.1.3 Oil absorption capacity (OAC)

The CLPs exhibited medium to high OAC ranging from 0.86 to 2.16 g/g, and values were similar within processing methods. Raw CLPs showed significantly lower OAC compared to leaves that were boiled prior to dehydration. A possible explanation for the presence of more intact hydrophilic groups in the raw sample compared to boiled samples that may have lost some hydrophilic compounds during extended boiling. Huang et al. (2020) reported similar values for 200 µm particle size Moringa powders. The authors found a decrease in oil holding capacity of superfine ground powders. Patil et al. (2020), however, observed higher OAC of 4.3 g/g for hot air‐dried amaranth. The relatively high OAC for CLPs makes them suitable for fat‐ and oil‐containing products and for flavor retention especially in baked products. Proper oil absorption is important for mouthfeel and flavor retention, but extremely high OAC can cause sogginess in deep‐fried or oil‐treated products. High OAC is beneficial for products that require oil, such as baked goods (Devisetti et al., 2016).

#### 3.1.4 Rehydration and dispersibility

Rehydration and dispersibility are important for the performance of powders in water‐based systems, as dispersibility determines how well the powder rehydrates without forming lumps (Mohajan et al., 2018). Heat treatment before drying improved rehydration and dispersibility in CLPs, with raw powder having the lowest and boiled samples showing the highest rehydration ratio. The hydrophilicity of the chemical constituents appears to be the key factor, as the processing that involved directly cooking in water resulted in the highest rehydration. These findings were comparable to and slightly higher than those reported for raw spinach powders (Waseem et al., 2021). Raw cowpea leaves showed the lowest dispersibility (45.55%), and samples steamed for 15 min had the highest (64.84%). A negative relationship was observed between dispersibility and several other parameters except WSI. The high OAC of powders from stir‐fry CLs significantly reduced dispersibility within the stir‐fry group. Dispersibility is also negatively correlated with bulk density, suggesting that the heavier the CLP relative to its volume, the slower it will disperse in water.

#### 3.1.5 Particle size distribution

**FIGURE 1:**
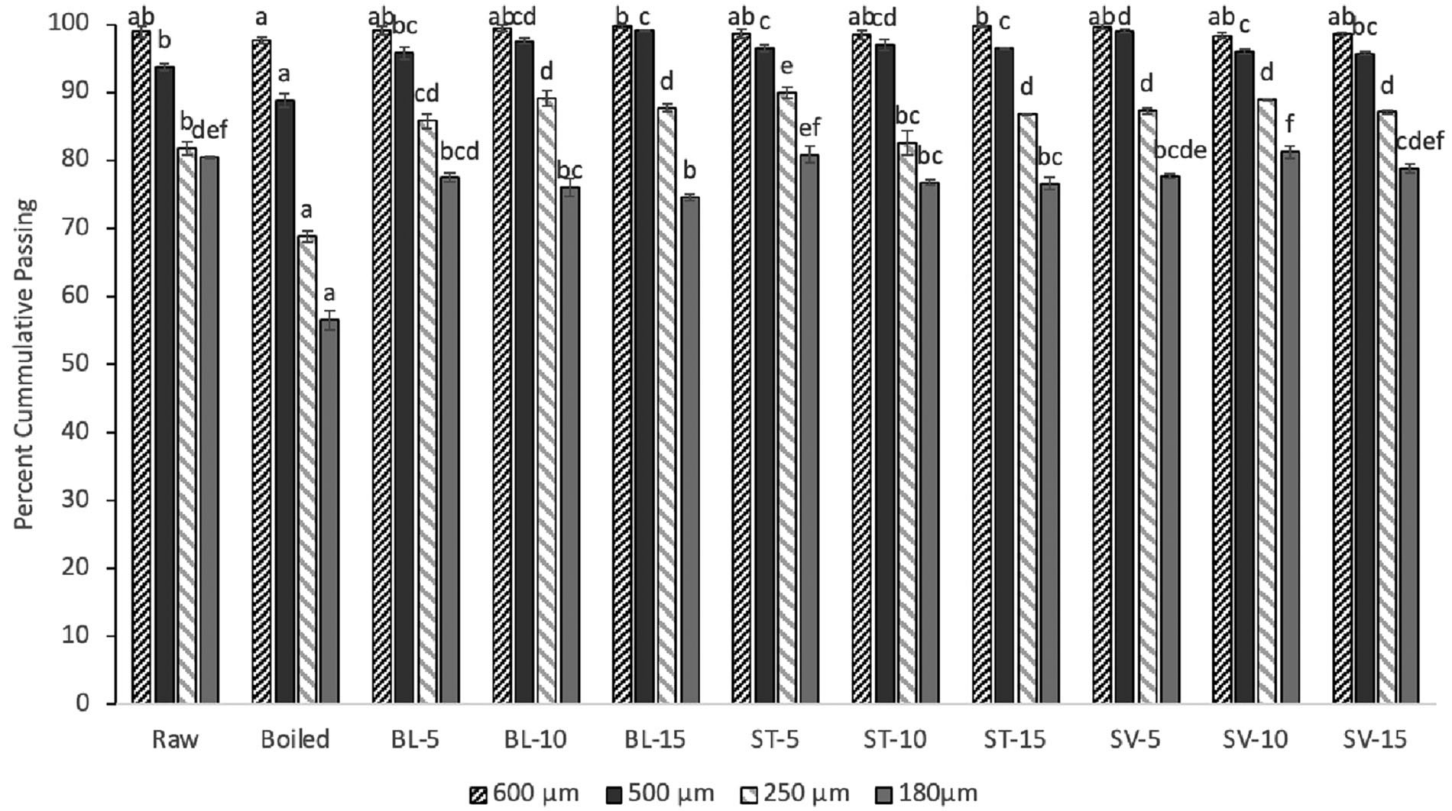
Particle size distribution of cowpea leaf powders prepared using different processing methods. Data are the mean of three replicates ± SD. Different superscripts within bars represent a significant difference at *p* ≤ 0.05. Raw, boiled, BL‐5, BL‐10, BL‐15, blanched for 5, 10, and 15 min, respectively; ST‐5, ST‐10, ST‐15, steamed for 5, 10, and 15 min, respectively; SV‐5, SV‐10, SV‐15, sous vide for 5, 10, and 15 min, respectively.

CLPs revealed fine particle sizes with over 98% and 90% of all samples (except for boiled cowpea leaves) passed through the 600 and 500 µm sieves, respectively (Figure 1). Samples from all 250 and 180 µm sieves had over 75% passing rate (except BCL), indicating an overall fineness in CLPs. Bas‐Bellver et al. (2022) reported similar findings for hot air‐dried cabbage and broccoli. They reported 90% of the powders obtained by dehydrating at 60°C passing through the at least 650 µm sieve and 50% through a minimum of 200 µm sieve. Boiled CP gave the lowest pass‐through fraction, suggesting a coarser particle size compared to other pretreatment methods of processing (Figure 1). Overall, sous‐vide CLPs were finer, followed by steamed and blanched CLPs. The exclusion of water during treatment is a factor in the fineness of cowpea leaves (Mythili et al., 2021). Initial high moisture content can cause rapid initial drying rates causing hardening of the outer layer but rubbery inner core with higher moisture content, making them difficult to mill (Bas‐Bellver et al., 2022; Gulati & Datta, 2015). Bas‐Bellver et al. (2022) reported finer particle size with higher drying temperatures for cabbage and the opposite for broccoli. The results for particle size are particularly important in the food processing industry when selecting a processing method to achieve specific particle size distribution.

### 3.2 Physicochemical properties

#### 3.2.1 L*, a*, and b* color

The results for analytical color are presented in Table 1. Each parameter represents the following: L*: lightness (0 = black, 100 = white), a*: green to red (−a* = green, +a* = red), blue to yellow (−b* = blue, +b* = yellow). The L*, a*, and b* values of fresh cowpea leaves were 47.61, −9.13, and 29.49, respectively. The CLP samples obtained after cooking and milling were somewhat dull being light green, except for the stir‐fried samples, which had a charcoal‐green appearance, confirmed by their extremely low L* values (13.76–14.10). The color a* values increased from −9.13 for raw CLs to −2.07 for 15 min stir‐fried CL (Table 2).

**TABLE 1:** Functional properties of cowpea leaf powders as affected by different processing methods.

Method	WAC (g/g)	WSI (%)	OAC (g/g)	Rehydration ratio (g/g)	Dispersibility (%)
Raw	3.52 ± 0.04 ^cde^	8.39 ± 0.26 ^b^	1.22 ± 0.10 ^a^	2.42 ± 0.21 ^a^	45.55 ± 0.17 ^b^
Boiled	4.26 ± 0.21 ^e^	3.74 ± 0.29 ^a^	2.16 ± 0.03 ^c^	6.99 ± 0.02 ^f^	49.45 ± 0.15 ^c^
Blanched					
5‐min	3.97 ± 0.50 ^de^	6.02 ± 0.50 ^a^	1.17 ± 0.00 ^ab^	3.11 ± 0.02 ^b^	52.04 ± 0.66 ^d^
10‐min	3.50 ± 0.26 ^cd^	5.17 ± 0.00 ^a^	1.21 ± 0.00 ^ab^	3.59 ± 0.09 ^c^	57.20 ± 0.43 ^f^
15‐min	3.63 ± 0.03 ^cde^	4.37 ± 0.13 ^a^	1.75 ± 0.09 ^abc^	3.59 ± 0.01 ^c^	42.64 ± 0.20 ^a^
Steamed					
5‐min	3.13 ± 0.04 ^bc^	6.05 ± 0.71 ^a^	1.34 ± 0.03 ^abc^	4.21 ± 0.01 ^d^	50.57 ± 0.82 ^cd^
10‐min	2.96 ± 0.07 ^bc^	10.35 ± 0.37 ^bcd^	1.20 ± 0.13 ^ab^	4.25 ± 0.00 ^d^	51.98 ± 0.74 ^d^
15‐min	2.43 ± 0.01 ^ab^	12.13 ± 0.00 ^e^	0.86 ± 0.00 ^ab^	4.99 ± 0.01 ^e^	64.84 ± 0.23 ^i^
Sous vide					
5‐min	3.22 ± 0.14 ^c^	11.07 ± 1.05 ^cde^	1.12 ± 0.14 ^ab^	4.30 ± 0.07 ^d^	60.90 ± 0.18 ^gh^
10‐min	3.50 ± 0.26 ^cd^	11.37 ± 0.12 ^de^	1.66 ± 0.72 ^abc^	4.01 ± 0.26 ^d^	55.03 ± 0.06 ^e^
15‐min	3.63 ± 0.03 ^cde^	12.03 ± 0.00 ^e^	1.19 ± 0.00 ^ab^	4.26 ± 0.08 ^d^	54.74 ± 0.51 ^e^
Stir‐fried					
5‐min	1.77 ± 0.05 ^a^	9.13 ± 0.42 ^bcd^	1.52 ± 0.01 ^abc^	4.16 ± 0.08 ^d^	60.03 ± 0.04 ^g^
10‐min	1.81 ± 0.13 ^a^	8.92 ± 1.24 ^bc^	1.34 ± 0.20 ^abc^	4.82 ± 0.04 ^e^	62.46 ± 0.06 ^h^
15‐min	1.92 ± 0.11 ^a^	10.17 ± 0.95 ^bcd^	0.99 ± 0.14 ^ab^	5.21 ± 0.07 ^e^	60.56 ± 0.08 ^g^

*Note*: Data are the mean of three replicates ± SD. Different superscripts within column represent a significant difference at *p* ≤ 0.05.

Abbreviations: OAC, oil absorption capacity; WAC, water absorption capacity; WSI, water‐solubility index.

**TABLE 2:** Effect of processing methods on the Tristimulus color values of cowpea leaf powders.

Method	L* value	a* value	b* value
Raw	47.61 ± 0.15 ^f^	−9.13 ± 0.01 ^a^	29.49 ± 0.00 ^fg^
Boiled	38.38 ± 0.78 ^b^	−3.21 ± 0.16 ^h^	25.49 ± 0.50 ^c^
Blanched			
5‐min	38.23 ± 0.31 ^b^	−7.25 ± 0.13 ^b^	27.71 ± 0.08 ^de^
10‐min	38.49 ± 0.30 ^b^	−6.50 ± 0.18 ^c^	27.47 ± 0.01 ^de^
15‐min	37.45 ± 0.16 ^b^	−6.00 ± 0.15 ^d^	26.70 ± 0.34 ^cd^
Steamed			
5‐min	44.13 ± 0.05 ^e^	−6.89 ± 0.17 ^bc^	31.15 ± 0.20 ^h^
10‐min	41.87 ± 0.06 ^cd^	−5.43 ± 0.06 ^ef^	28.13 ± 0.46 ^def^
15‐min	40.60 ± 0.12 ^c^	−3.07 ± 0.01 ^h^	28.62 ± 0.22 ^ef^
Sous vide			
5‐min	42.06 ± 0.26 ^d^	−5.69 ± 0.06 ^de^	28.86 ± 0.13 ^ef^
10‐min	44.01 ± 0.13 ^e^	−4.87 ± 0.01 ^g^	31.21 ± 0.12 ^h^
15‐min	44.37 ± 0.18 ^e^	−5.09 ± 0.06 ^fg^	30.69 ± 0.23 ^gh^
Stir‐fried			
5‐min	14.1 ± 0.17 ^a^	−2.79 ± 0.15 ^hi^	9.77 ± 0.81 ^b^
10‐min	14.37 ± 0.80 ^a^	−2.38 ± 0.09 ^ij^	7.05 ± 0.63 ^a^
15‐min	13.76 ± 0.04 ^a^	−2.07 ± 0.05 ^j^	8.79 ± 0.18 ^b^

*Note*: Data are the mean of three replicates ± SD. Different superscripts within column represent a significant difference at *p* ≤ 0.05.

The loss of green color observed in cowpea leaf during cooking is related to the loss of chlorophyll and its conversion to pheophytin, resulting in a dark and brown appearance during heat treatment. The results for stir‐fried samples contradict the findings of Akdaş and Bakkalbaş (2017), who found an increase in the green color intensity of stir‐fried kale. This difference is potentially due to differences in sample matrices and cooking times, impacting the degree of dehydration and color. Browning and green color loss increased with longer cooking times in cowpea leaves except for sous vide. Among water‐based cooking methods, boiled cowpea leaves exhibit the least greenness (a* = −3.21). Turkmen et al. (2006) found chlorophyll retention ranging from 19% to 100%, in vegetables during cooking. The degree of retention was dependent on the vegetable type and cooking method.

Our results were consistent with previous studies that examined the effects of different cooking methods on various vegetables, such as broccoli, African pumpkin leaves, and zucchini (Mashiane et al., 2021). These studies suggested that prolonged cooking times resulted in increased discoloration, loss of green color, and a downward trend in a* values. They found that the color difference was much greater for boiled samples than steamed and raw samples. Drastic color changes in cooked samples can be attributed to changes in intercellular water‐filled spaces. Fried and boiled samples resulted in lower b* values compared to microwave cooking (Danowska‐Oziewicz et al., 2020; Mashiane et al., 2021).

Blanched CL samples retained higher color intensity, followed by steamed, sous‐vide, boiled, and fried samples. However, sous vide showed the slowest rate of color loss between 5 and 15 min. In terms of lightness, sous vide exhibited the highest L* values (42.06–44.37), surpassing blanched (37.45–38.49), whereas fried samples had the lowest values. These findings are consistent with Ilic et al. (2022), who observed more intense coloring in steamed eggplant compared to sous vide. The dark green color in fried CLP may be due to intracellular air replacement with oil, leading to rapid water loss and significant chlorophyll and carotenoid loss (Akdaş & Bakkalbaş, 2017) (Table 2).

**TABLE 3:** Physicochemical properties of cowpea leaf powders.

Method	Moisture (g/100 g)	Water activity, *a*_w_	pH	Protein (g/100 g) d.b.
Raw	6.34 ± 0.47 ^ab^	0.53 ± 0.00 ^fg^	6.29 ± 0.02 ^g^	17.68 ± 0.02 ^cd^
Boiled	6.79 ± 0.28 ^b^	0.49 ± 0.01 ^defg^	6.15 ± 0.01 ^de^	18.67 ± 0.10 ^d^
Blanched				
5‐min	6.00 ± 0.74 ^ab^	0.49 ± 0.02 ^defg^	6.26 ± 0.06 ^fg^	20.45 ± 0.02 ^e^
10‐min	6.45 ± 2.95 ^ab^	0.52 ± 0.01 ^efg^	6.29 ± 0.01 ^g^	20.80 ± 0.02 ^e^
15‐min	6.82 ± 0.14 ^b^	0.55 ± 0.00 ^g^	6.53 ± 0.02 ^h^	20.34 ± 0.00 ^e^
Steamed				
5‐min	5.01 ± 0.13 ^ab^	0.49 ± 0.00 ^defg^	5.93 ± 0.01 ^bc^	17.64 ± 0.03 ^cd^
10‐min	6.88 ± 0.16 ^b^	0.49 ± 0.00 ^defg^	5.79 ± 0.00 ^a^	17.01 ± 1.15 ^c^
15‐min	5.23 ± 0.45 ^ab^	0.47 ± 0.02 ^cde^	5.86 ± 0.01 ^ab^	18.47 ± 0.05 ^d^
Sous vide				
5‐min	6.66 ± 0.15 ^b^	0.26 ± 0.01 ^a^	6.21 ± 0.01 ^ef^	17.75 ± 0.03 ^cd^
10‐min	6.30 ± 0.01 ^ab^	0.36 ± 0.00 ^b^	6.14 ± 0.00 ^de^	17.95 ± 0.05 ^cd^
15‐min	5.66 ± 0.13 ^ab^	0.33 ± 0.00 ^b^	6.09 ± 0.01 ^d^	17.85 ± 0.08 ^cd^
Stir‐fried				
5‐min	3.69 ± 0.23 ^ab^	0.43 ± 0.01 ^c^	6.12 ± 0.01 ^d^	11.92 ± 0.02 ^b^
10‐min	3.65 ± 0.03 ^ab^	0.43 ± 0.03 ^c^	6.15 ± 0.01 ^de^	10.79 ± 0.08 ^ab^
15‐min	3.26 ± 0.04 ^a^	0.46 ± 0.01 ^cd^	5.94 ± 0.01 ^c^	10.51 ± 0.03 ^a^

*Note*: Data are the mean of three replicates ± SD. Different superscripts within column represent a significant difference at *p* ≤ 0.05.

Abbreviation: d.b., dry weight basis.

#### 3.2.2 Moisture and water activity

The moisture content of CLPs ranged from 3.26 g/100 g (stir‐fried for 15 min) to 6.82 g/100 g (blanched for 15 min) (Table 3). The lowest water activity (0.26–0.36) was achieved for the sous‐vide group, followed by the stir‐fry category (0.43–0.46). The highest water activity (0.53) was obtained for raw cowpea leaves. The low water activity observed in sous‐vide samples could be attributed the removal of water during cooking combined with no direct contact with water in sous‐vide cooking. Mythili et al. (2021) reported similar higher water activity in raw cauliflower powder compared to hot water blanched and steamed samples, showing the least water activity. The trends observed in cooked CLPs compared to raw samples were also similar to those reported for blanched papaya leaves (Raja et al., 2019).

Despite some differences among processing methods, the moisture content of the CLPs observed in this study is considered desirable and well below the minimum standard of 15% for food powders. Water activity is a good indicator of the shelf stability of food powders, which is important for predicting biochemical reactions, microbial activity, food spoilage, and shelf‐life. The CLPs in our study can be classified as low‐moisture foods (*a*_w_ < 0.85), making them more likely to be resistant to microbial activity and shelf‐stable. The pretreated powders could be well suited for use in instant and ready‐to‐eat food products.

#### 3.2.3 pH

Acidity can influence taste, texture, and nutritional value (Wickramasinghe et al., 2020). The pH of CLPs in this study indicated a slightly acidic concentration. Raw CLP had a pH of 6.29, whereas boiled CLP was 6.15. Similar trends were observed for blanched and sous‐vide samples with minimal differences. Steamed samples had the lowest pH values, ranging from 5.79 to 5.94. Wickramasinghe et al. (2020) reported a decrease in the pH of raw Moringa leaves after steaming. This variation could be attributed to the different water‐soluble acids present in Moringa and cowpea leaves, which may be lost in steam or during cooling.

#### 3.2.4 Protein

Protein content was high in CLPs (10.51–20.80 g/100 g), raw cowpea leaves exhibited lower protein content (17.68 g/100 g) compared to blanched CLP, which had the highest protein content. No significant differences were recorded within the blanched and sous‐vide groups timewise. CL that was steamed for 15 min had higher protein content than 5‐ and 10‐min blanched leaves. Conversely, CL that was fried for 15 min showed the least amount of protein within the stir‐fry group. The protein content of sous vide and steamed CL was similar to those of raw and boiled samples.

The protein content of raw CLP was lower than reported for the Ghana CL variety (24%–35%) and blanched sun‐dried Kenyan variety (33%) (Dakora & Belane, 2019; Owade et al., 2022). The use of a nitrogen conversion factor of 4.5, as opposed to the traditional 6.25, to account for non‐protein nitrogen in leafy vegetables may contribute to the observed differences. Our results align with the protein content in spinach leaf powder (19.18%) reported by Waseem et al. (2021). The effects of blanching/boiling in our study were consistent with Owade et al. (2022), who found higher protein content in blanched sun‐dried cowpea leaves compared to the unblanched ones. Ayele et al. (2022) also noted increased protein content in cassava leaves after blanching. Conversely, Kshirsagar et al. (2017) reported reduced protein content with blanching and maximum retention with steaming. However, Mepba et al. (2007) found that cooking had no significant impact on the protein content of leafy green vegetables.

Stir‐fried CLPs showed the least amount of protein content (11.92 g/100 g for 5 min to 10.52 g/100 g for 15 min of stir‐fried samples). Protein content decreased with longer cooking times in oil. Such reductions are typical in frying processes, where higher temperatures and rapid moisture loss lead to protein denaturation and loss. The extent of these losses can vary depending on factors such as food matrix, oil type, and temperature (Sahin & Sumnu, 2006). These findings were supported by previous research, where it was reported that stir‐fried broccoli retained the least total soluble protein compared to steamed broccoli (Yuan et al., 2009). However, the literature presents conflicting views on the impact of frying on protein content, with some studies reporting minimal effects or even increased protein content (Oke et al., 2018).

#### 3.2.5 Mineral and heavy metal profile

The mineral and heavy metal profiles of cowpea leave powder as result of different processing methods are presented in Tables 4 and 5, respectively. Raw CLP had significant amounts of major minerals, including iron, calcium, and potassium; 25 mg/100 g iron, 1690 mg/100 g calcium, 1760 mg/100 g, 649.5 mg/100 g potassium, 235 mg/100 g phosphorus, 649.5 mg/100 g magnesium, and 22.50 µg/100 g selenium. CLPs have appreciable amounts of zinc (2.75–4.15 mg/100 g) for 5 and 15 min blanched, respectively. The length of blanch time and treatment type significantly impacted calcium, magnesium, and sodium. Raw cowpea leaves had sodium content ranging from 920 to 635 mg/100 g and were significantly reduced by 75% in boiled and blanched samples. Direct contact with water led to greater reduction than in steamed and sous‐vide samples and to a lesser extent in stir‐fried samples. Overall, most minerals increased with cooking time within the blanched, steamed, and sous‐vide categories but decreased with time for stir‐fry cooking. Calcium, for example, significantly reduced to 995 mg/100 g in stir‐fry samples. Stir‐frying cowpea leaves before dehydration significantly reduced most minerals.

**TABLE 4:** Effect of processing methods on the mineral profile of cowpea leave powder (mg/100 g except selenium as µg/100 g).

Method	Iron	Zinc	Calcium	Sodium	Potassium	Magnesium	Phosphorous	Selenium
Raw	25.04 ± 0.64 ^f^	3.3 ± 0.00 ^bcde^	1690 ± 42.43 ^ef^	920 ± 14.14 ^f^	1765 ± 63.64 ^fg^	649.5 ± 27.58 ^d^	235 ± 5.66 ^cde^	22.50 ± 5.32 ^a^
Boiled	28.1 ± 0.28 ^g^	3.60 ± 0.85 ^cde^	1440 ± 14.14 ^d^	200 ± 0.89 ^b^	630 ± 0.00 ^b^	377 ± 0.00 ^a^	210 ± 1.41 ^b^	26.50 ± 7.13 ^a^
Blanched								
5‐min	21.9 ± 1.27 ^e^	2.75 ± 0.07 ^abc^	1155 ± 7.07 ^b^	125 ± 7.07 ^a^	555 ± 7.07 ^ab^	462.5 ± 0.71 ^abc^	220 ± 0.00 ^bc^	11.50 ± 2.40 ^a^
10‐min	26.65 ± 0.07 ^fg^	3.50 ± 00 ^cde^	1325 ± 35.36 ^c^	185 ± 7.07 ^b^	575 ± 7.07 ^ab^	409.5 ± 4.95 ^ab^	260 ± 1.02 ^fg^	10.00 ± 2.96 ^a^
15‐min	26.15 ± 0.49 ^fg^	3.05 ± 0.14 ^abcd^	1490 ± 56.57 ^d^	210 ± 0.65 ^b^	515 ± 35.36 ^a^	392.5 ± 17.68 ^ab^	240 ± 14.14 ^bcdef^	9.00 ± 1.67 ^a^
Steamed								
5‐min	20.85 ± 0.49 ^de^	3.90 ± 0.07 ^de^	1690 ± 14.14 ^ef^	745 ± 14.14 ^d^	1630 ± 28.28 ^e^	444 ± 14.14 ^abc^	250 ± 0.00 ^defg^	19.5 ± 3.39 ^a^
10‐min	18.80 ± 0.01 ^cd^	4.10 ± 0.07 ^e^	1760 ± 14.14 ^f^	730 ± 7.07 ^d^	1695 ± 21.21 ^ef^	502.5 ± 95.46 ^abcd^	255 ± 7.07 ^fg^	10.00 ± 1.95 ^a^
15‐min	18.15 ± 0.64 ^c^	4.15 ± 0.00 ^e^	1740 ± 28.28 ^f^	720 ± 7.07 ^d^	1505 ± 21.21 ^d^	474.5 ± 118.09 ^abc^	270 ± 2.02 ^g^	10 ± 0.21 ^a^
Sous vide								
5‐min	19.6 ± 0.57 ^cd^	3.25 ± 0.07 ^bcde^	1620 ± 0.00 ^e^	935 ± 7.07 ^f^	1785 ± 21.21 ^fg^	544 ± 5.66 ^abcd^	230 ± 0.12 ^bcd^	14 ± 1.5 ^a^
10‐min	18.7 ± 0.14 ^c^	3.4 0 ± 0.00 ^bcde^	1670 ± 14.14 ^ef^	905 ± 14.14 ^f^	1835 ± 7.10 ^g^	551.5 ± 2.12 ^bcd^	245 ± 7.07 ^cdef^	9.5 ± 0.11 ^a^
15‐min	17.9 ± 0.14 ^c^	3.25 ± 0.07 ^bcde^	1725 ± 21.21 ^f^	865 ± 7.07 ^e^	1795 ± 35.36 ^fg^	604.5 ± 6.36 ^cd^	230 ± 0.24 ^bcd^	10.5 ± 0.95 ^a^
Stir‐fried								
5‐min	13.4 ± 0.14 ^b^	2.80 ± 0.00 ^abc^	1225 ± 21.21 ^bc^	845 ± 14.14 ^e^	1050 ± 14.14 ^c^	479.5 ± 3.54 ^abc^	170 ± 0.00 ^a^	18 ± 3.86 ^a^
10‐min	10.8 ± 0.28 ^a^	2.55 ± 0.00 ^ab^	1190 ± 28.28 ^b^	750 ± 7.07 ^d^	1000 ± 14.14 ^c^	534 ± 3.54 ^abcd^	150 ± 0.00 ^a^	12.5 ± 0.91 ^a^
15‐min	12.7 ± 0.71 ^ab^	2.30 ± 0.07 ^a^	995 ± 7.07 ^a^	635 ± 7.07 ^c^	1075 ± 21.21 ^c^	507.5 ± 4.95 ^abcd^	155 ± 7.07 ^a^	15 ± 2.09 ^a^

*Note*: Data are the mean of three replicates ± SD. Different superscripts within row represent a significant difference at *p* ≤ 0.05.

**TABLE 5:** Effect of processing methods on the heavy metal profiles of cowpea leave powder (mg/kg).

Method	Aluminum	Arsenic	Lead	Cadmium	Chromium	Nickel	Cobalt	Silicon	Titanium
Raw	78.5 ± 12.02 ^bc^	0.42 ± 0.10 ^a^	0.15 ± 0.00 ^a^	0.05 ± 0.00 ^a^	7.55 ± 0.04 ^a^	3.45 ± 0.2 ^bc^	0.31 ± 0.02 ^c^	13.6 ± 1.6 ^a^	2.74 ± 0.27 ^b^
Boiled	110.5 ± 4.42 ^ef^	0.26 ± 0.00 ^a^	0.12 ± 0.00 ^a^	0.05 ± 0.00 ^a^	2.4 ± 0.00 ^a^	1.3 ± 0.1 ^a^	0.15 ± 0.02 ^ab^	87.45 ± 0.1 ^a^	1.28 ± 0.08 ^a^
Blanched									
5‐min	81.00 ± 1.41 ^bcd^	0.25 ± 0.00 ^a^	0.10 ± 0.00 ^a^	0.02 ± 0.00 ^a^	5.55 ± 0.63 ^a^	6.55 ± 0.1 ^d^	0.26 ± 0.01 ^bc^	57.45 ± 9.0 ^a^	0.44 ± 0.05 ^a^
10‐min	134.5 ± 21.92 ^f^	0.3 ± 0.00 ^a^	0.14 ± 0.00 ^a^	0.03 ± 0.00 ^a^	1.95 ± 0.01 ^a^	1.05 ± 0.1 ^a^	0.12 ± 0.02 ^a^	31.2 ± 4.1 ^a^	0.54 ± 0.07 ^a^
15‐min	108. ± 0.00 ^def^	0.25 ± 0.1 ^a^	0.22 ± 0.1 ^a^	0.02 ± 0.00 ^a^	1.25 ± 0.04 ^a^	0.8 ± 0.1 ^a^	0.11 ± 0.01 ^a^	57.7 ± 24.2 ^a^	0.92 ± 0.55 ^a^
Steamed									
5‐min	69.5 ± 3.54 ^bc^	0.27 ± 0.1 ^a^	0.1 ± 0.00 ^a^	0.03 ± 0.00 ^a^	7.55 ± 0.04 ^a^	5.1 ± 0.00 ^cd^	0.22 ± 0.01 ^abc^	74.75 ± 7.1 ^a^	0.64 ± 0.13 ^a^
10‐min	36.5 ± 3.54 ^a^	0.35 ± 0.1 ^a^	0.12 ± 0.00 ^a^	0.02 ± 0.00 ^a^	1.3 ± 0.06 ^a^	0.9 ± 0.4 ^a^	0.13 ± 0.05 ^a^	83.25 ± 32.5 ^a^	0.94 ± 0.54 ^a^
15‐min	53.00 ± 4.42 ^ab^	0.34 ± 0.01 ^a^	0.1 ± 0.01 ^a^	0.02 ± 0.00 ^a^	2.9 ± 0.24 ^a^	2.05 ± 0.18 ^a^	0.15 ± 0.09 ^ab^	65.7 ± 1.3 ^a^	0.66 ± 0.01 ^a^
Sous vide									
5‐min	94.5 ± 0.71 ^cde^	0.44 ± 0.00 ^a^	0.09 ± 0.00 ^a^	0.02 ± 0.00 ^a^	2.1 ± 0.00 ^a^	1.1 ± 0.01 ^a^	0.14 ± 0.01 ^a^	43.7 ± 0.54 ^a^	0.89 ± 0.00 ^a^
10‐min	87 ± 1.41 ^cde^	0.32 ± 0.01 ^a^	0.05 ± 0.00 ^a^	0.02 ± 0.00 ^a^	1.00 ± 0.06 ^a^	0.95 ± 0.05 ^a^	0.17 ± 0.00 ^ab^	57.05 ± 3.87 ^a^	0.64 ± 0.24 ^a^
15‐min	85.5 ± 0.71 ^cde^	0.27 ± 0.00 ^a^	0.05 ± 0.00 ^a^	0.02 ± 0.00 ^a^	2.5 ± 0.01 ^a^	1.9 ± 0.00 ^ab^	0.17 ± 0.00 ^ab^	64.35 ± 0.5 ^a^	0.65 ± 0.07 ^a^
Stir‐fried									
5‐min	37.5 ± 2.12 ^a^	0.36 ± 0.00 ^a^	0.09 ± 0.00 ^a^	0.02 ± 0.00 ^a^	0.6 ± 0.00 ^a^	0.6 ± 0.01 ^a^	0.12 ± 0.00 ^a^	68.4 ± 3.54 ^a^	0.97 ± 0.04 ^a^
10‐min	35.0 ± 0.00 ^a^	0.35 ± 0.00 ^a^	0.05 ± 0.00 ^a^	0.02 ± 0.00 ^a^	0.5 ± 0.00 ^a^	0.5 ± 0.00 ^a^	0.11 ± 0.01 ^a^	51.85 ± 2.3 ^a^	0.62 ± 0.04 ^a^
15‐min	53.5 ± 2.12 ^ab^	0.31 ± 0.00 ^a^	0.08 ± 0.00 ^a^	0.02 ± 0.00 ^a^	0.75 ± 0.02 ^a^	0.55 ± 0.01 ^a^	0.13 ± 0.00 ^a^	62.8 ± 4.06 ^a^	0.9 ± 0.19 ^a^

*Note*: Data are the mean of three replicates ± SD. Different superscripts within row represent a significant difference at *p* ≤ 0.05.

No significant differences were found among cooking methods for selenium content, ranging from 9 µg/100 g (for 15 min blanched) to 26.5 µg/100 g (boiled) samples. Selenium contents were comparable to concentrations (up to 6 µg/100 g fresh weight) in spinach (US Department of Agriculture Agricultural Research Service, 2019). CLPs in this study had lower calcium, potassium, zinc, magnesium, and phosphorus contents compared to a study by Kruger (2020) for a cowpea variety from South Africa. In addition to the influence of location, it is also important to note that the authors used shorter cooking times and included the cooking water to freeze‐dry cowpea leaf samples. Freeze‐drying is the gold standard in the food industry due to optimal nutrient retention in comparison to any other method of dehydration. The CLP processed from dried raw leaves had an iron content of 25 mg/100 g. This aligns with the findings of Kruger (2020) (29.3 mg/100 g iron on a dry matter basis) for leaves blanched for two min and freeze‐dried with residual cooking water.

The iron content in boiled cowpea leaves was significantly higher (28.1 mg/100 g) than in raw CLP in this study. This contrasts with the findings of Owade et al. (2022), who found that iron content decreased by more than 70% and 50% when blanched sun‐drying and blanched shade‐drying were used, respectively. The elevated iron content in boiled cowpea leaves represents contamination, from stainless cooking pots or non‐distilled water (Park & Brittin, 1997). This trend was evident in the decreasing pattern observed for iron content for cooking methods that did not have the cowpea leaves having direct contact with water or stainless cooking pots, as seen in the sous‐vide and steaming methods. Madaki and Asquo (2021) observed a significant increase in iron content of rice that was boiled in stainless steel.

Contamination of vegetables with heavy metals can come from soil, water, cooking pots, or during processing. Contamination from cultivation on abandoned refuse dumpsites and during cooking in aluminum pans is common in developing countries such as Ghana (Twumasi et al., 2016; Weidenhamer et al., 2017). The heavy metals of public health concern, like lead and cadmium, have FAO/World Health Organization (WHO) permissible limits for food at 0.1 and 0.2 mg/kg, respectively (FAO/WHO, 2019). Most heavy and light metals, including lead in CLPs, were below the WHO permissible limits except for aluminum. Minimal to no effect of cooking on heavy metals was observed in this study. Cooking may decrease, increase, or have no impact on the concentration of heavy metals. The type of effect is dependent on the type of heavy metal, food material as well as processing method (Beihrozi et al., 2023; Inobeme et al., 2020). The findings in this study are consistent with other studies who reported insignificant effect of various cooking methods on heavy metals (Kananke et al., 2015; Perelo et al., 2008). Other studies observed lower concentrations of heavy metals for methods like boiling, steaming, and sous vide due to water‐solubility, leaching and heating effects on metals such as lead, arsenic, and cadmium in various foods, including vegetables (Diyarov et al., 2022). Lee et al. (2019) observed decreased levels of heavy metals in noodles that were boiled for 3 min.

With regards to aluminum, significant differences were observed between cooking methods, especially among the raw, boiled, blanched, and fried samples. Aluminum concentrations were generally higher in blanched and boiled samples compared to raw, and most of the steamed, sous‐vide, and especially stir‐fried samples. Literature on the effects of cooking on aluminum content is limited. Contrary to our finding, Lee et al. (2019) reported a decrease in aluminum content in noodles that were boiled between 3 and 10 min due to possible dissolution in cooking water. A reduction in other metals, such as copper during frying of bush meat, has been reported (Kobia et al., 2016). The significant loss of aluminum during stir‐frying could be explained by two phenomena: (1) water loss at high temperatures (Inobeme et al., 2020) and/or (2) the formation of different compounds and complexes during heat at high temperatures (Kobia et al., 2016). The relatively higher levels of aluminum in blanched and boiled samples suggest a relationship between direct contact with water and, cooking pot, combined with the effect of heat. Shamloo et al. (2023) reported the leaching of different heavy metals to varied degrees into summer vegetables and meat distilled toxic‐metal‐free water in diverse types of pots made from varied materials, including stainless steel pots. The concentration and the degree of leaching of heavy metal were greater for pots that were old and increased with cooking time. The concentration of aluminum reported by the authors for stainless steel pot was significantly low (0.018–0.087 mg/kg) for foods boiled up to 4 h. Ojezele et al. (2016), however, reported high aluminum levels of 289 and 298 mg/kg in rice that was cooked for 20 min in old and new stainless pots, respectively. Differences are likely due to variation in the rates of uptake by different food materials.

Aluminum is known to interfere with iron absorption (Colomina & Peris‐Sampedro, 2017). The aluminum concentration in this study was 35–134 mg/kg. The contamination of food with heavy metals, such as aluminum, is common in many developing countries and poses a serious public health concern. Common sources of contamination include soil, water, and cooking utensils (Jaishankar et al., 2014; Munir et al., 2022).

The concentrations of arsenic were 0.27–0.44 mg/kg and levels were not impacted by heat application or cooking method (Table 5). Arsenic is readily taken up by leafy green crops (McBride, 2013). There are currently no general guidelines by the WHO and similar commissions on arsenic levels in food, but the United States Food and Drug Administration established limits of 100 ppb for inorganic arsenic in infant cereal. Therefore, we can conclude that arsenic levels are concerning. Food preparation methods, such as soaking, can significantly reduce arsenic content in certain foods, but boiling, for example, in water containing arsenic, may increase levels above toxicological standards (Devesa et al., 2008). Arsenic exposure carries health risks, including cancer, skin lesions, cardiovascular disease, and diabetes. Therefore, cowpea leaves containing lower levels of arsenic should be consumed or used for food fortification.

## 4 CONCLUSION

The current study demonstrates that fresh cowpea leaves can be processed into powder with stable functional and physical properties after pre‐cooking with the methods investigated, except stir‐frying, which had flowability challenges due to oil content. The results revealed diverse physicochemical, mineral, heavy metal, and protein profiles due to species differences. Various cooking times and methods showed varied effects on physicochemical properties, protein, and mineral content, depending on the processing parameter, or nutrient analyzed, but insignificant effects on heavy metal concentrations. All processing methods except stir‐frying showed high retention of protein and minerals. Sous vide and steaming imparted a brighter appearance to cowpea leaves, enhancing their visual appeal. CLPs exhibited unique and favorable functional properties, suitable for incorporation into water and oil systems, particularly in ready‐to‐eat foods. Despite their high protein content and minerals like calcium, magnesium, and potassium, the powders contained elevated aluminum levels and arsenic levels. Sous vide and steaming were recommended for optimal mineral retention, with steaming being more cost‐effective. Blanching, boiling, and stir‐frying reduced undesirable heavy metal levels more effectively. These powders offer opportunities for enriching food products and ingredients with functional properties, protein, and micronutrients. Additional analysis of amino acid profile, protein quality, antinutrients, and mineral bioavailability is also of nutritional relevance and would provide information on the efficacy of the powders in addressing nutritional deficiencies. Further investigations into processing and growing conditions in Ghana are warranted to mitigate aluminum and heavy metal concentrations in CLPs.

## AUTHOR CONTRIBUTIONS

**Makafui Borbi**: Conceptualization; investigation; writing—original draft; methodology; writing—review and editing; formal analysis; data curation. **Lorraine Weatherspoon**: Conceptualization; supervision; writing—review and editing. **Jason Wiesinger**: Writing—review and editing; formal analysis; methodology; data curation; validation. **Jose Jackson**: Writing—review and editing; supervision; conceptualization. **Raymond Glahn**: Methodology. **Leslie Bourquin**: Conceptualization; supervision. **Kirk Dolan**: conceptualization; writing—review and editing; supervision.

## CONFLICT OF INTEREST STATEMENT

The authors have no conflicts of interest to declare.

## Supporting information

Table S1 Correlation among different parameters for cowpea leaf powders.
