# Supplementary material for: Effects of different processing methods on the functional, nutritional, and physicochemical profiles of cowpea leaf powder
Source: J Food Sci. 2024 Dec 15;89(12):8715–29. doi: 10.1111/1750-3841.17569 (PMC11673565; doi:10.1111/1750-3841.17569)
Supplement: Supplementary file 1 — Table S1 Correlation among different parameters for cowpea leaf powders. [file JFDS-89-8715-s001.docx]

**Supplementary material**

**Table 1: Correlation between different parameters for cowpea leaf powders**

|  |  |  |  |  |  |  |  |  |  |  |  |  |  |  |  |
| --- | --- | --- | --- | --- | --- | --- | --- | --- | --- | --- | --- | --- | --- | --- | --- |
|  |  | 1 | 2 | 3 | 4 | 5 | 6 | 7 | 8 | 9 | 10 | 11 | 12 | 13 | 14 |
| 1 | MC |  |  |  |  |  |  |  |  |  |  |  |  |  |  |
| 2 | Aw | .06 |  |  |  |  |  |  |  |  |  |  |  |  |  |
| 3 | pH | .32 | .14 |  |  |  |  |  |  |  |  |  |  |  |  |
| 4 | L | .75** | -.01 | .08 |  |  |  |  |  |  |  |  |  |  |  |
| 5 | a | -.56** | -.23 | -.40* | -.72** |  |  |  |  |  |  |  |  |  |  |
| 6 | b | .74** | -.02 | .08 | .99** | -.70** |  |  |  |  |  |  |  |  |  |
| 7 | Bulk density | .35 | .78** | -.07 | .39* | -.36 | .41* |  |  |  |  |  |  |  |  |
| 8 | WAC | .79** | .11 | .44* | .77** | -.63** | .78** | .33 |  |  |  |  |  |  |  |
| 9 | WSI | -.24 | -.67** | -.52** | -.04 | .29 | -.05 | -.56** | -.44* |  |  |  |  |  |  |
| 10 | OAC | .24 | .16 | .37 | .02 | .08 | .02 | .01 | .35 | -.53** |  |  |  |  |  |
| 11 | Rehydration | -.17 | -.13 | -.41* | -.3 | .76** | -.29 | -.04 | -.13 | -.03 | .31 |  |  |  |  |
| 12 | Dispersibility | -.53** | -.50** | -.47* | -.50** | .65** | -.47* | -.43* | -.67** | .60** | -.47* | .32 |  |  |  |
| 13 | Protein | .77** | .2 | .36 | .85** | -.67** | .89** | .56** | .85** | -.37 | .14 | -.29 | -.51** |  |  |
| 14 | % passing(fineness) | -.25 | -.24 | -.09 | .55** | -.53* | .77** | -.25 | -.48* | .52* | -.61** | -.79** | .17 | -.2 |  |
